# Medical Society of the State of New York

**Published:** 1899-03

**Authors:** 


### MEDICAL SOCIETY OF THE STATE OF NEW YORK.

THE ninety-third annual meeting of the Medical Society of the State of New York was held in the City Hall, Albany, January 31 and February 1 and 2, 1899. From a scientific and social standpoint it must be regarded as one of the most successful in the history of the society; the attendance was perhaps larger than in recent years and especially on the morning of the first day, when the large hall was filled to its full capacity. The attendance continued good throughout the sessions and the debates and discussions were numerous and spirited. The utmost good feeling pervaded its proceedings and at no time was there any spirit of antagonism or sting of rebuke discoverable. As presiding officer, Dr. John O. Roe, of Rochester, was in the best mien and presided with a natural grace and dignity which added much to the success of the meeting. He listened attentively to the reading of papers and was firm but courteous in his rulings, thus showing a proper respect and decorum, unhappily not always observed by presiding officers. In his inaugural address he called attention to the very prosperous condition of the society, as shown by the program and by the fact that at present the membership amounted to 708. In calling attention to the legislative work he referred to the dispensary bill, showed up the preposterous pretensions of the anti-vivisectionists in their efforts to pass their bill, and the benefits to be derived from the passing of the pure food bills, now before the legislature.

The committee reports were unusually interesting, especially those of the committees on hygiene and legislation and the board of medical examiners.

Dr. Lewi, of the state board of medical examiners, stated that of 809 applicants coming before the board, representing the society, 29.9 per cent. were refused. Among the New York graduates there was but one rejected paper for every three students. The board wisely suggested a change in the examination papers and in the grouping of subjects.

Dr. Hopkins, of Buffalo, read the report of the committee on hygiene, which was enthusiastically received and much commented on. Hardly has such an interesting and instructive report ever been made by a committee, and its importance was sufficient to warrant its separate publication and distribution by the unanimous vote of the society.

From the chairman's report of the committee on legislation, Dr. Van Fleet proved that the committee had had its hands comfortably full during the last session of the legislature, and the committee would be in excellent condition to testify in favor of biennial, preferably triennial, sessions of the legislature, as now advocated by many. The report stated that more than forty bills had been presented to the legislature, none, however, at the instigation of the state society. Among the bills defeated by the committee were the Sullivan dispensary bill, the bill for regulating the practice of osteopathy, the anti-vaccination bill, and the bills regarding premature burial and regulating the practice of midwifery.

The special committee's report on the revision of the U. S. Pharmacopeia, by Dr. Eli H. Long, of Buffalo, was adopted by the society and the committee continued.

The business committee, under the chairmanship of Dr. Willis G. Macdonald, prepared a very full and interesting program, so voluminous, in fact, that on Wednesday the society was divided into a surgical and a medical section—an occurrence which we trust will not be necessary again. The meetings of the state medical society ought to be neutral ground where the specialists of all departments can meet the so-called general practitioner, and discussions ensue which shall prove that after all the science of medicine is a unit in which all integral parts are dependent upon each other for autonomy. Special societies are numerous enough and there are sections enough in our national societies without making the state society lose its distinctive qualities as a society of general medicine in its broadest sense. Make but one section, Mr. Chairman, lasting three days, put some of the important papers on the afternoon of the third day, allow a full discussion, and if over-burdened with papers, advise the authors to prepare a résumé with permission to print in full in the transactions.

To the tact and good management of the business committee the program was closely adhered to and when the first day's session was ended the program for the day had been exhausted. Among the many papers presented all showed careful study and research and were of an unusually high scientific character. Buffalo, as usual, had its full quota of papers that attracted much favorable comment and discussion. On the first day papers were read by Dr. William G. Bissell on the Transmission of disease by the use of second-hand clothing; by Dr. Ernest Wende, on The dangers of the long-tube nursing-bottle; by Dr. William C. Krauss, on The close relation between the nasal and cranial cavities and between nasal and cranial diseases; by Dr. H. R. Hopkins, on Hygienic camps—with a discussion participated in by Dr. William Warren Potter; by Dr. John H. Pryor, on The relation of the consumptive to the state; and a lantern exhibition on Some interesting cases of skin diseases, by Dr. Grover W. Wende. Dr. Herman Mynter and other Buffalo members took an active part in the discussion of several papers and it seemed for a time as if a Buffalo medical society were holding its meeting at Albany. On the second day, Dr. Roswell Park read a paper on A further study into the nature and frequency of cancer, which was discussed by Dr. H. R. Gaylord, assistant director of the state laboratory.

On Wednesday afternoon the sections met together and listened to an address on Some problems associated with typhoid fever, by Dr. William Osler, of Baltimore, and another on The disinfection of the alimentary canal, by Dr. Abraham Jacobi, of New York. Both papers were well received and much enjoyed.

Wednesday evening in the Senate Chamber, Dr. John O. Roe, delivered the President's address, taking for his subject The relation of medicine to civilisation. Dr. Roe handled his subject in a masterful manner, and showed that the general advance of scientific knowledge had resulted not from progress in any one department, but from simultaneous discoveries in each, each contributing to the advancement of all the others. The annual banquet followed immediately at the Kenmore.

Thursday was devoted to the reports of the committees appointed by the chair and the reading of papers. The committee on president's address reported that it was in favor of a bill being prepared, which would make it a penal offence for applicants at charitable medical institutions to receive gratuitous medical services at these places when they are able to pay. The committee also urged the legislative committee to make an effort to restore the appropriation of $5,000 for the maintenance of the state medical library.

Dr. Suiter called attention to the serious hampering of the work of the State Board of Health by the very meager appropriation made by the state, *i. e.*, about $35,000, as compared with $200,000 appropriated for the fish and game commission.

The election of officers resulted as follows: President, Dr. Willis G. Macdonald, of Albany; vice-president, Dr. John Gerin, of Auburn; secretary, Dr. Frederic C. Curtis, of Albany; treasurer, Dr. Charles H. Porter, of Albany. For honorary membership, Drs. W. W. Keen, of Philadelphia and Maurice H. Richardson, of Boston.

In the distribution of committeeships Buffalo fared very well, with Dr. H. R. Hopkins, chairman of the committee on hygiene; Dr. Ernest Wende, on the legislative committee; Dr. William C. Krauss, on the committee on prize essays and Dr. William Warren Potter, on the publication committee.

The nominations to fill vacancies on the State Board of Medical Examiners consists of Drs. A. Walter Suiter, of Herkimer; George R. Fowler, of New York; H. D. Wey, of Elmira, and Daniel Lewis, of New York.

Next to Buffalo our Rochester brethren were fully represented both on the floor and in committees. With Dr. Roe in the president's chair, Dr. E. B. Angell on the business committee, and papers read by Drs. L. A. Weigel, H. T. Williams, J. W. Whitbeck, E. W. Mulligan, the medical prestige of our sister city was fully sustained.

